# Emodin reverses gemcitabine resistance in pancreatic cancer cells via the mitochondrial apoptosis pathway *in vitro*

**DOI:** 10.3892/ijo.2011.1285

**Published:** 2011-12-07

**Authors:** DIAN-LEI LIU, HEQI BU, HONG LI, HUI CHEN, HONG-CHUN GUO, ZHAO-HONG WANG, HONG-FEI TONG, ZHONG-LIN NI, HAI-BIN LIU, SHENG-ZHANG LIN

**Affiliations:** 1 Department of Surgery, The Second Affiliated Hospital of Wenzhou Medical College, No. 109, West Xue-yuan Road, Wenzhou 325027; 2 Wenzhou Vocational College of Science and Technology, Wenzhou Liuhong Road No. 1000, Wenzhou 325000, P.R. China

**Keywords:** emodin, reverse, pancreatic cancer, gemcitabine-resistance

## Abstract

Gemcitabine resistance is a common problem of pancreatic cancer chemotherapy, and how to reverse it plays an important role in the treatment of pancreatic cancer. This study investigated the effect of emodin on the gemcitabine-resistant pancreatic cancer cell line SW1990/Gem, and explored the potential mechanism of its action. SW1990/Gem was obtained by culture of the pancreatic cancer cell line SW1990 *in vitro* by intermittently increasing the concentration of gemcitabine in the culture medium for 10 months, observing the morphology using inverted microscopy. SW1990/Gem cells were pretreated with emodin (10 μM) for different periods followed by treatment with gemcitabine (20 μM) for 48 h; cell proliferation was tested by MTT assay. SW1990/Gem cells were treated by emodin with different concentrations for 48 h, cell apoptosis was detected by flow cytometry (FCM). The expression of gene and protein, such as MDR-1 (P-gp), NF-κB, Bcl-2, Bax, cytochrome-C (cytosol), caspase-9 and -3 were measured by RT-PCR and Western blotting. The function of P-gp in SW1990/Gem cells was checked by FCM. The results showed that the SW1990/Gem cells changed greatly in morphology and the resistance index was 48.63. Emodin promoted cell apoptosis of the gemcitabine-resistant cell line SW1990/Gem in a dose-dependent manner. Emodin enhanced the SW1990/Gem cell sensitivity to gemcitabine in a time-dependent manner. Emodin monotherapy or combination with gemcitabine both decreased the gene and protein expression levels of MDR-1 (P-gp), NF-κB and Bcl-2 and inhibited the function of P-gp, but increased the expression levels of Bax, cytochrome-C (cytosol), caspase-9 and -3, and promoted cell apoptosis. This demonstrated that emodin had a reversing effect on the gemcitabine-resistant cell line SW1990/Gem, possibly via decreasing the function of P-gp and activating the mitochondrial apoptosis pathway *in vitro*.

## Introduction

Pancreatic cancer is a common and highly malignant tumor of the digestive system. In recent years, the global morbidity and mortality of pancreatic cancer is increasing. In Europe, new cases of pancreatic cancer in 2006 were 60,000, and 59,000 cases of death, ranking fifth in cancer mortality in Europe (1). In the United States, new cases were 37,170 in 2006, and 33,370 cases of death; in 2010, new cases were 43140, ranking fourth in cancer mortality in the United States (2,3). Surgical resection is the only hope to improve survival rate, but the vast majority of pancreatic cancer patients are diagnosed at advanced stage, and prognosis is poor, the average 5-year survival rate is less than 5% (3). In addition to surgery, chemotherapy is still an important means to treat advanced pancreatic cancer, prevent post-surgical recurrence, prolong survival time and improve life quality. Gemcitabine is currently the best first-line chemotherapy to treat advanced pancreatic cancer (4), but due to acquired or intrinsic drug resistance of pancreatic cancer cells (5,6), effect of using gemcitabine to treat pancreatic cancer is still not ideal (7), which causes patients treated with gemcitabine to suffer from side effects of chemotherapy without good treatment. Thus, it is particularly important to find a drug that can reverse the drug-resistance and enhance the treatment of gemcitabine. Emodin (6-methyl-1,3,8-tanthragallol), which is the main active monomer separated from Rheum, Polygonum, buckthorn and senna, is a tyrosine kinase II inhibitor, it has effects of anti-microbial activity (8–11), anti-inflammatory (12,13), immunosuppression (14), anti-tumor (15,16), and activity in protection of liver cells (17). Our team previously found emodin could promote apoptosis (16), and established the gemcitabine-resistance cell line SW1990/Gem, we confirmed that emodin could increase the sensibility of resistance cell line to gemcitabine through inhibiting the expression of NF-κB (18). However, it is still unclear whether emodin can reverse the gemcitabine-resistant human pancreatic cancer cells, thus this study mainly investigates the effect of emodin reversing the gemcitabine-resistance on SW1990/Gem cell line, and its possible mechanisms.

## Materials and methods

### Chemicals and cell lines

The followings were purchased: Emodin (purity >98%), MTT, DMSO (Sigma), gemcitabine (Eli Lilly and Co.), FBS, trypsin containing EDTA, Roswell Park Memorial Institute-1640 (RPMI-1640) (Gibco), BCA protein assay kit (Pik-day Institute of Biotechnology), Rhodamine123 (Rh123), Annexin V-FITC/PI apoptosis detection kit (Biological Development Co., Ltd. Nanjing KGI), RNA extraction kit (Life Technologies Co.); cDNAfirst strand synthesis kit (Fermentas), 2X Taq PCR MasterMix (Tiangen), P-gp, NF-κB, Bcl-2, Bax, cytochrome-C antibodies (Santa Cruz). Drug preparation: Emodin was dissolved in DMSO as a 0.2 mM stock solution and stored at −20°C. DMSO concentration <0.1% (it has no effect on cell proliferation when concentration was <0.1%). Gemcitabine is reconstituted as a 0.02 mM stock solution in sterile saline. Cell culture: The human pancreatic cancer cell line SW1990 was purchased from the American Type Culture Collection. Cells were cultured in RPMI-1640 medium, supplemented with 10% fetal calf serum (FCS), in a humidified atmosphere of 5% CO_2_ and 95% air at 37°C.

### Establishment of gemcitabine-resistant human pancreatic cancer cell line SW1990/Gem

Gemcitabine-resistant pancreatic cancer cell line SW1990/Gem was obtained by culture of pancreatic cancer cell line SW1990 *in vitro* with intermittently increasing the concentration of gencitabine in the culture medium for 10 months. After cultivating SW1990 cells with different concentrations of gemcitabine for 1 week, we checked the cell death conditions and chose the concentration of median lethal dose (LD_80_) (which could kill 80% cells) as the initial concentration to cultivate the resistant cell line. Cells were cultivated in this medium for 48 h, and then incubated in RPMI-1640 medium without drugs. When cells grew stably and entered the logarithmic growth phase, they were passaged twice, and exposed to gemcitabine in double LD_80_ concentration, after nine concentration gradients and ~10 months of cultivation, they were finally incubated in RPMI-1640 medium without drugs for 2 months.

### Morphological assay of gemcitabine-resistant cell line SW1990/Gem

Two lines of logarithmic phase SW1990/Gem and SW1990 cells were incubated in a 6-well plate at a density of 100,000 cells per well for 2 days, and were observed by optical microscope (Nikon, TS100), and then were collected separately and fixed for electron microscopic observation of cell ultra-structures.

### Sensitivity analysis of SW1990/Gem to gemcitabine

The logarithmic phase SW1990/Gem and SW1990 cells were incubated in a 96-well plate at a density of 4,000 cells per well. Cells were cultured in different concentrations (20, 40, 80 and 160 μM) of gemcitabine for 48 h after they adhered. Each group had 6-wells. The supernatant was discarded and 20 μl MTT (5 mg/ml) was added with 180 μl medium to each well, 4 h later the culture medium was removed and 150 μl DMSO was added to each well. The plate was shaken by microplate shaker for 10 min and the absorbance (A) of samples was measured at 490 nm by automatic enzyme-linked immunosorbent assay. The experiment was repeated three times. The drug inhibition of cells was calculated by the following formula: Inhibition = 1-dosing group A/control group A × 100%. Data was graphed on a semi-logarithmic curve with drug concentrations plotted on the x-axis and cell inhibitions on the y-axis. SPSS software was used to calculate the 50% inhibitory inhibition (IC_50_) (19) and the resistance index (RI). RI = IC_50_ of resistance cell line/IC_50_ of the sensitive cell line.

### Effect of gemcitabine on SW1990/Gem proliferation after pretreatment with emodin

SW1990/Gem cells were incubated in a 96-well plate at a density of 4,000 cells per well overnight. Cells were pretreated with low emodin (10 μM) for different periods (12, 24, 36, 48 and 60 h) and then incubated with gemcitabine for 48 h. Emodin was not added to the control group, and it was directly incubated in gemcitabine for 48 h. The supernatant was discarded and MTT (5 mg/ml) was added, 4 h later the culture medium was removed and 150 μl DMSO was added to each well. The plate was shaken by a microplate shaker for 10 min and absorbance (A) of samples were measured. Each group had 6-wells. The experiment was repeated three times, and the cell viability was calculated.

### Effect of emodin on SW1990/Gem cell apoptosis

The logarithmic phase SW1990/Gem cells were incubated in a 6-well plate (4×10^5^/well), treated with different concentrations of emodin (10, 20, 40, 80 and 160 μM) and the control group when cells were 80% confluent. Forty-eight hours later, cells were collected and centrifuged at 1000 rpm/min for 5 min. Cells were washed with cold PBS 3 times and resuspended with 500 μl binding buffer, then 5 μl Annexin V-FITC was added, mixed, and cultured in the dark for 5 min, adding 10 μl PI for 5 min. The fluorescence of cells was measured by flow a cytometer at 488/530 nm. The experiment was repeated three times, and cells were analyzed with Cell Quest software.

### Protein expression of MDR-1 (P-gp), NF-κB, Bcl-2, Bax, cytochrome-C (cytosol), caspase-9 and -3 were detected in SW1990/Gem and SW1990 by Western blotting

SW1990/Gem and SW1990 cells were collected, the cytoplasmic protein was evaluated with the mitochondrial/cytoplasmic protein isolation kit to detect cytochrome-C levels according to the instructions. Cells were lysed with RIPA, centrifuged at 12000 rpm/min, collecting the supernatant. Protein concentrations were measured with BCA kit, then the amount of protein was equaled, in 10% SDS-PAGE electrophoresis, PVDF membrane was transferred, then blocked with 5% skimmed milk powder, incubated with antibodies at 4°C overnight, then washed with TBST and incubated with secondary antibodies for 2 h, after washing with TBST and coloring with ECL, the membranes were exposed to X-rays. The experiment was repeated three times.

### Protein expression of P-gp, NF-κB, Bcl-2, Bax, cytochrome-C, caspase-9 and -3 in SW1990/Gem after treatment with emodin and/or gemcitabine by Western blotting

SW1990/Gem was treated with emodin (10 μM) and gemcitabine (20 μM) alone or together for 48 h, cytoplasmic protein was gathered with the mitochondrial/cytoplasmic protein isolation kit to detect cytochrome-C levels according to the instructions. The other steps were as above.

### Analysis of MDR-1 (P-gp), NF-κB, Bcl-2, Bax, cytochrome-C, caspase-9 and -3 in SW1990/Gem after treatment with emodin and/or gemcitabine by reverse transcription-PCR

After cultivating SW1990/Gem cells treated with emodin (10 μM) and gemcitabine (20 μM) alone or combined for 48 h, cells were lysed by TRIzol and RNA was extracted, then the content of RNA was measured by UV spectrophotometer at 260 nm. cDNA under the instruction of the first Fermentas cDNA strand synthesis kit was synthesized (Tiangen 2X Taq PCR MasterMix instruction) the PCR amplification conditions were: MDR-1, 94°C 30 sec, 57°C 30 sec, 72°C 30 sec, 30 cycles; NF-κB, 94°C 30 sec, 54°C 30 sec, 72°C 20 sec, 30 cycles; Bcl-2, 94°C 20 sec, 58°C 20 sec, 72°C 20 sec, 35 cycles; Bax, 94°C 30 sec, 57°C 30 sec, 72°C 20 sec, 30 cycles; cytochrome-C, 94°C 30 sec, 60°C 30 sec, 72°C 30 sec, 35 cycles; caspase-9, 94°C 30 sec, 56°C 30 sec, 72°C 30 sec, 35 cycles; caspase-3, 94°C 30 sec, 57°C 30 sec, 72°C 30 sec, 35 cycles; GAPDH, 94°C 30 sec, 54°C 30 sec, 72°C 20 sec, 25 cycles. GAPDH was used as an internal control. Product (5-μl) was added to the 1.5% agarose gel electrophoresis and images were taken. The RT-PCR sequences of primers and the size of the sequences are shown in Table I.

### Analysis of P-gp function in SW1990/Gem by Rhodamine123 (Rh123) efflux experiment (flow cytometric analysis)

After SW1990/Gem cells were incubated with emodin and gemcitabine alone or combination for 48 h, they were resuspended in medium (1×10^6^/ml), then Rhodamine 123 staining solution 10 μg/ml was added and cultured in an incubator with 37°C 5% CO_2_ for 30 min, centrifuged and washed with medium twice, resuspended in medium and incubated in the incubator for 120 min, centrifuged again and washed with PBS twice, measured by flow cytometry at 488/530 nm. This experiment was repeated three times.

### Statistical analysis

Data were expressed as the mean ± SD and evaluated by SPSS16.0. The differences were considered to be statistically significant at P<0.05.

## Results

### Biological properties of the gemcitabine-resistant cell line SW1990/Gem and sensitivity testing

**Figure 1:**
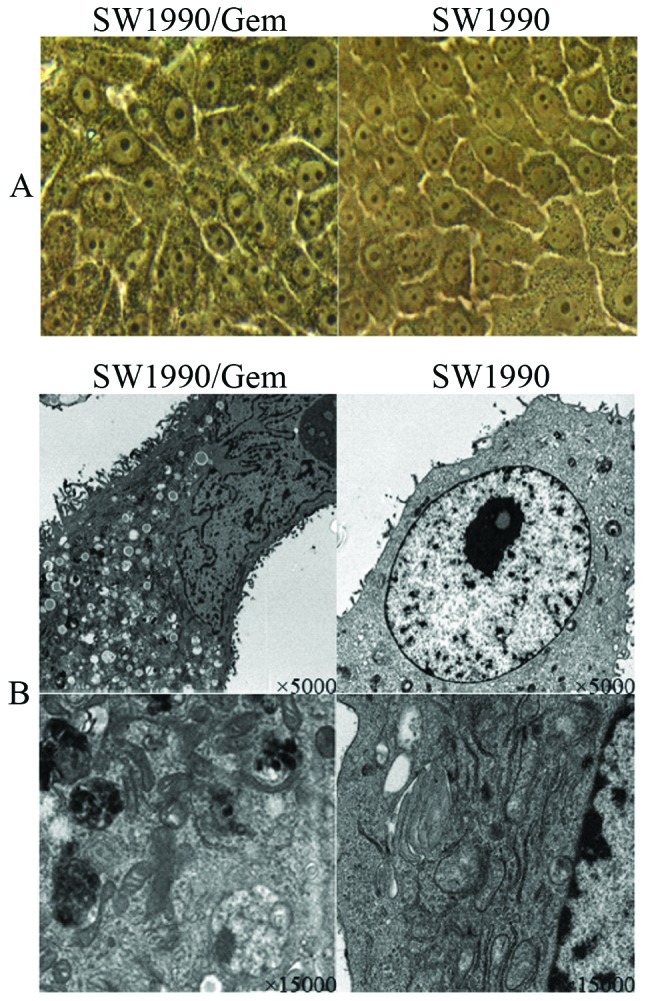
The changes of biological properties. (A) Compared with SW1990, the volume of SW1990/Gem cells increased, SW1990/Gem cell size varied and the granular substances increased, cells with more nucleoli increased. (B) Compared with SW1990, microvilli at the surface of the SW1990/Gem cell membranes increased, the SW1990/Gem cell surface area increased, cell organs in the cytoplasm increased, mitochondria cristaes were disordered, vacuoles were found in the cell matrix, endoplasmic reticulum and the vacuole structures in the cytoplasm were increased.

We achieved the stable passage resistance cell line after 10 months via *in vitro* culture, after incubation without drugs for 2 months, compared with parental cell line SW1990, the SW1990/Gem changed significant in morphology. Under light microscope (x400) (Fig. 1A), the volume of SW1990/Gem cells increased, was different in size and the granular substances increased, SW1990/Gem cells with more nucleoli increased. Under electron microscopy (×5,000 and ×15,000) (Fig. 1B), microvilli at the surface of the SW1990/Gem cell membranes increased, the SW1990/Gem cell surface area increased, cell organelles in the cytoplasm increased, mitochondria cristaes were disordered, vacuoles were also found in the SW1990/Gem cell matrix, endoplasmic reticulum and the vacuole structures in cytoplasm were increased. The gemcitabine-resistant cell line SW1990/Gem 50% inhibitory concentration (IC_50_) was 1267.53±26.78 μM, the resistance index was 48.63. The resistant cell line SW1990/Gem showed significant resistance to gemcitabine.

### After SW1990/Gem cells were pretreated with emodin, the sensitivity to gemcitabine was significantly enhanced

**Figure 2:**
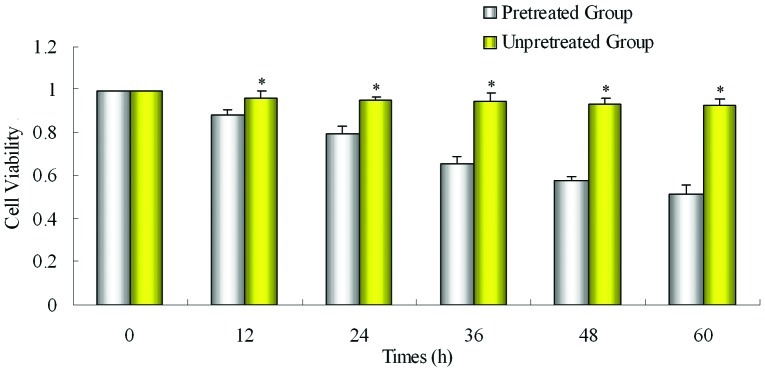
SW1990/Gem cells pretreated with emodin at 10 μM for different periods (12, 24, 36, 48 and 60 h), and then treated with gemcitabine for 48 h (20 μM), cell proliferation was analyzed by MTT, the group pretreated with emodin decreased the cell viability of SW1990/Gem in a time-dependent manner, compared with unpretreated group, ^*^P<0.05.

Pretreated group was pretreated with emodin and then treated with gemcitabine for 48 h, unpretreated group was treated with only gemcitabine for 48 h. Compared with the unpretreated group, the inhibiting effect of gemcitabine on proliferation of gemcitabine-resistant cell line SW1990/Gem was significantly enhanced in pretreated group (Fig. 2).

### Promoting effect of emodin on SW1990/Gem cell apoptosis

**Figure 3:**
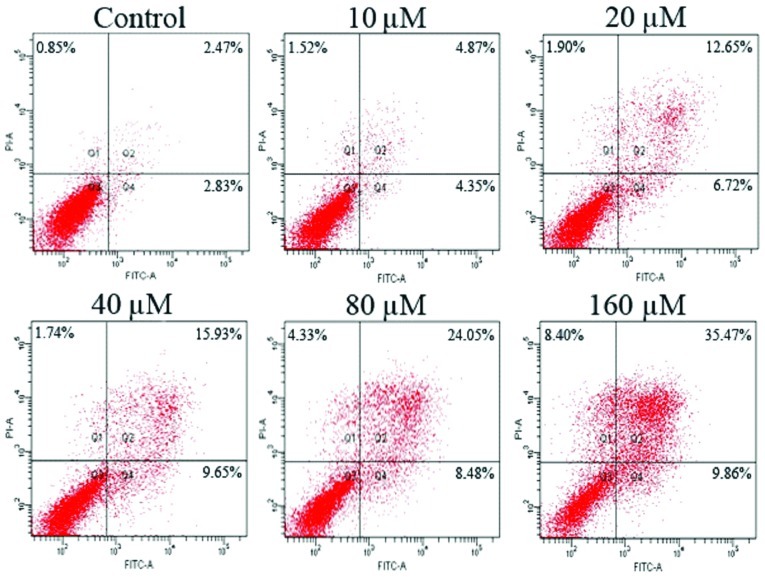
SW1990/Gem cells were treated with different concentrations of emodin (10, 20, 40, 80 and 160 μM) for 48 h, cell apoptosis was analyzed by FCM. Emodin promoted SW1990/Gem cells apopotosis in a dose-dependent manner.

SW1990/Gem cells were treated with different concentrations of emodin (10, 20, 40, 80 and 160 μM) for 48 h, FCM was applied to analyze cell apoptosis. As Fig. 3 showed that emodin promoted cell apoptosis of gemcitabine-resistant cell line SW1990/Gem in a dose-dependent manner.

### Different protein expression of the P-gp, NF-κB, Bcl-2, Bax, cytochrome-C (cytosol), caspase-9 and -3 in SW1990/Gem and SW1990 cells

**Figure 4:**
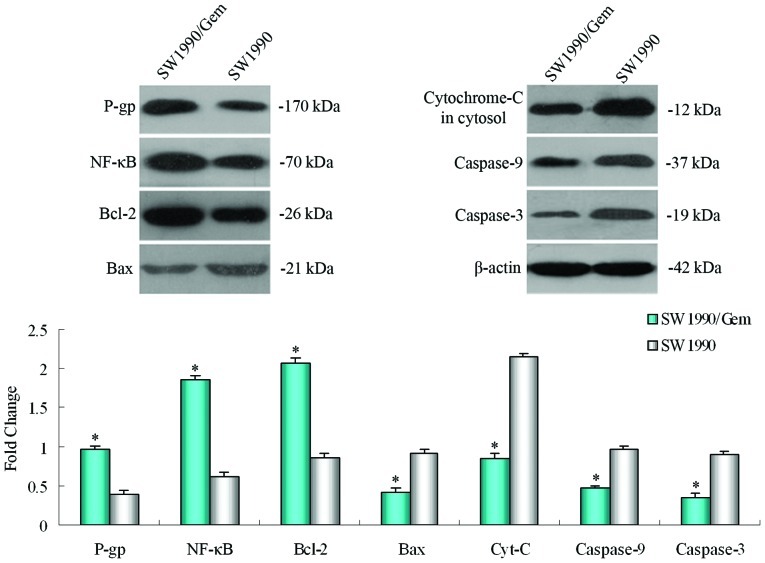
The protein expression was detected by Western blotting. Increased expression was seen of P-gp, NF-κB and Bcl-2 in SW1990/Gem, decreased expression of Bax, cytochrome-C (cytosol), caspase-9 and -3 in SW1990/Gem. Compared with SW1990, ^*^P<0.05.

Determined the basal expression of protein P-gp, NF-κB, Bcl-2, Bax, cytochrome-C (cytosol), caspase-9 and-3 in SW1990/Gem cells and SW1990 cells by Western blotting. Compared with parental cell line SW1990, the expression of multidrug-resistance gene encoding protein P-gp, apoptosis regulatory protein in mitochondrial pathway Bcl-2 and the NF-κB increased in the SW1990/Gem cells, while the expression of Bax regulated by Bcl-2 in mitochondrial pathway, cytochrome-C (cytosol), the caspase-9 and -3 of caspase family decreased obviously (Fig. 4).

### Effect of emodin on NF-κB and its related proteins in SW1990/Gem cells

**Figure 5:**
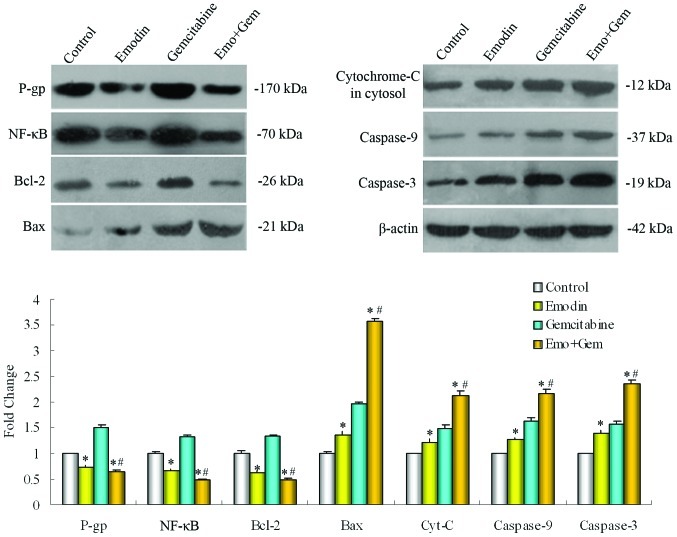
The protein expression was detected by Western blotting. Emodin alone and combined with gemcitabine down-regulated the protein expression of P-pg, NF-κB and Bcl-2, and up-regualted the expression of cytochrome-C (cytosol), caspase-9 and -3. Compared with control group, ^*^P<0.05; compared with gemcitabine group, ^#^P<0.05.

SW1990/Gem cells were treated with emodin (10 μM) alone or combined with gemcitabine (20 μM) for 48 h. The expression of protein was measured by Western blotting. As shown in Fig. 5, emodin alone or combined with gemcitabine down-reguglated the expression of NF-κB in SW1990/Gem cells, and then decreased the NF-κB regulated multidrug resistance protein P-gp and the Bcl-2 in mitochondrial pathway, then increased the expression of Bax and cytochrome-C (cytosol) regulated by Bcl-2 in mitochondrial pathway. Caspase cascade was triggered followed by the apoptosis of pancreatic cancer cells, thus, inhibition of pancreatic cancer growth and promotion of apoptosis occurred.

### Effect of emodin on the mRNA of NF-κB and its regulated genes in SW1990/Gem cells

**Figure 6:**
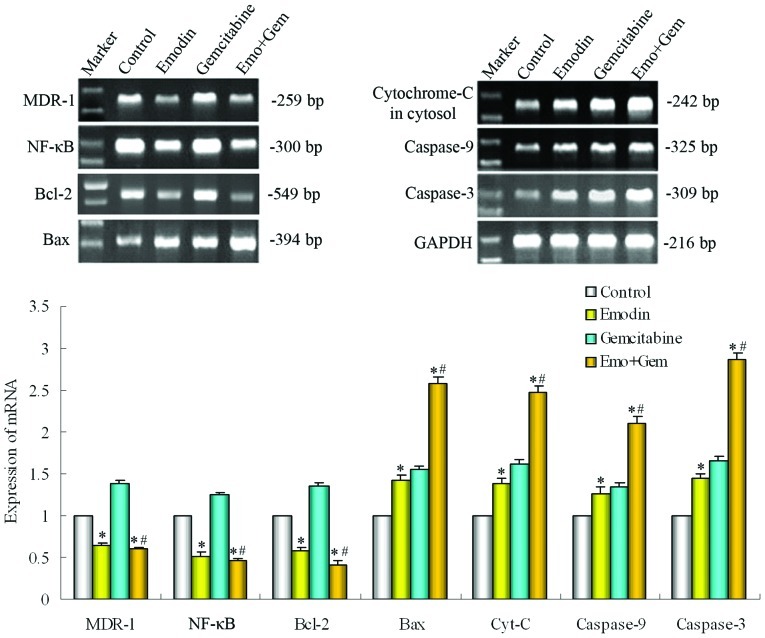
The mRNA expression was detected by RT-PCR, emodin alone and combined with gemcitabine could down-regulate the expression of MDR-1, NF-κB and Bcl-2, up-regulate the expression of Bax, cytochrome-C (cytosol), caspase-9 and -3. Compared with control group, ^*^P<0.05; compared with gemcitabine group, ^#^P<0.05.

The SW1990/Gem cells were treated with emodin (10 μM) alone or combined with gemcitabine (20 μM) for 48 h. The expression of gene was measured by PT-PCR. As shown in Fig. 6, emodin alone or combined with gemcitabine both down-regulated the expression of NF-κB, then down-regulated the gene expression of MDR-1 and Bcl-2, but up-regulated the expression of Bax, cytochrome-C, caspase-9 and -3, these were in line with the proteins change and validated the effect of emodin further.

### Effect of emodin on the the function of P-gp in SW1990/Gem cells

**Figure 7:**
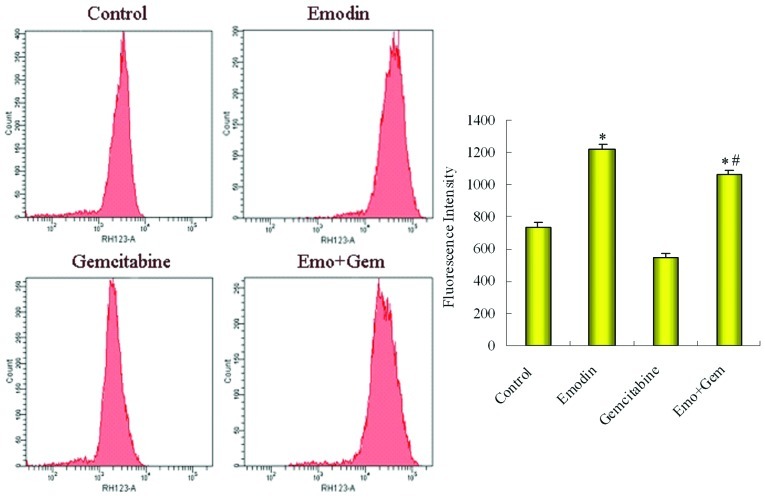
The P-gp function in SW1990/Gem was observed by Rhodamine123 (Rh123) efflux experiment (flow cytometric analysis). Emodin combined with gemcitabine up-regulated the fluorescence intensity. Compared with the control group, ^*^P<0.05; compared with gemcitabine group, ^#^P<0.05.

The level of rhodamine efflux function was determined by FCM after SW1990/Gem cells were treated with emodin alone and then combined with gemcitabine. As shown in Fig. 7, the fluorescence intensity was 734.62±25.74 in control group cells, 1225.28±28.55 in emodin-treated group, 545.23±28.27 in gemcitabine-treated group cells and 1068.44±22.85 in the combined group. Compared with control group, emodin decreased the function of P-gp; compared with gemcitabine-treated group, the combined group also decreased function of P-gp.

## Discussion

Pancreatic cancer is a common gastrointestinal tumor, because most patients are diagnosed at advanced stage, the surgical resection rate is low and chemo-therapy is the most critical treatment. Gemcitabine is the standard chemotherapeutic agent for advanced pancreatic cancer, but in clinical work the treatment effect is not ideal due to the increase of gemcitabine-induced drug resistance. The rate of 5-year overall survival is less than 5% and drug resistance is the main reason for the failure of the chemo-treatment in pancreatic cancer (6). According to reports, the mechanism of resistance to chemotherapy may be associated with increased drug cellular efflux by overexpressed P-gp encoded by multidrug resistance gene (MDR-1) and deregulated expression of anti-apoptotic or pro-apoptotic molecules (20,21), may also be associated with multidrug resistance-associated protein (MRP) (22).

As a traditional Chinese medicine, emodin not only has anti-tumor effect (15), but also can enhance the anti-tumor effect of the chemo-therapy drugs (16,23,24). It has been reported that emodin sensitizes the ovarian cancer cells and the gallbladder cancer cells to chemotherapeutic agents (25,26). However, reports on whether emodin can reverse the chemotherapeutic drug resistance in pancreatic cancer are rare. Our group first reported that emodin sensitized resistant cell to gemcitabine through inhibiting the expression of NF-κB in SW1990/Gem cells (18). In this study, we established the gemcitabine-resistant cell line SW1990/Gem with intermittently increasing the concentration of gemcitabine in the culture medium for 10 months, then calculated the resistance index and observed cell morphology. In the follow-up, SW1990/Gem cells were pretreated with emodin (10 μM) for different periods and then treated with gemcitabine (20 μM) for 48 h, cell viability was detected by MTT. The results showed that inhibition effect of gemcitabine on proliferation of drug-resistant cell line SW1990/Gem was significantly enhanced after cells were pretreated with emodin. FCM results showed that emodin could promote cell apoptosis in drug-resistant cell line SW1990/Gem. Western blotting detected the basal expression of P-gp, NF-κB, Bcl-2, Bax, cytochrome-C (cytosol), caspase-9 and -3 in SW1990/Gem cells and SW1990 cells, it was found that compared with parental cell line SW1990, the expression of P-gp, NF-κB and Bcl-2 was increased in the SW1990/Gem cells, while the expression of Bax, cytochrome-C, caspase-9 and -3 was decreased. Based on this, we furher investigated the potential molecule mechanism of reversing the resistance effect of gemcitabine-resistant pancreantic cancer cell line SW1990/Gem by emodin, possibly it is via decreasing the function of P-gp and the mitochondrial apoptosis pathway.

P-gp encoded by multidrug resistance gene-1 (MDR-1) is a kind of transmembrane glycoprotein, belongs to transporter protein superfamily ABC and has the ATP-dependent drug efflux function (22). P-gp can induce drug resistance due to decrease in the cellular chemotherapeutic drug under effective concentration through pumping the chemotherapeutic agents out of the cells against the concentration (27). Therefore, MDR-1/P-pg plays an important role in tumor chemotherapeutic resistance (28,29). According to reports, nuclear transcription factor NF-κB induces resistance of tumor cells through down-regulating the expression of MDR-1 mRNA (30). Other reports stated that the decrease of P-gp in expression and function reversed the chemotherapeutic resistance in breast cell line MCF-7, through the inhibition of MDR-1 expression induced by the decrease of expression and activity of NF-κB (31). In this study, the levels of gene and protein of the NF-κB and MDR-1 (P-gp) were decreased both in emodin group and in gemcitabine group. Rhodamine efflux experiments indicated that the function of P-gp was decreased both in emodin group and combination group. The decrease of expression and function of P-gp directly increased the intracellular drug concentration, and this may be partly the response to the reversion of the gemcitabine-resistance in pancreatic cancer.

Apoptosis defection is another important reason for drug resistance. Chemotherapeutic agents, as one of the major treatments of cancer, kill tumor cells mainly through inducing apoptosis, and the defection of apoptosis is one of the important reasons for drug-resistance due to insensitive of the tumor to chemotherapy (32).

Bcl-2 protein family is very important in apoptosis, the anti-apoptotic protein Bcl-2 and pro-apoptotic protein Bax are the major members in this family. Bcl-2 and Bax also play very important roles in mitochondrial pathway (33), the down-regulation of Bcl-2 and the up-regulation of Bax can induce the release of cytochrome-C (cytosol) from mitochondria, trigger the activity of caspase-3 and -9 and finally cause cell apoptosis (34). This study verified that the expression of Bcl-2 in SW1990/Gem was significantly higher than SW1990, but the expression of Bax and cytochrome-C were significantly lower suggesting that the mitochondrial receptor pathway may be involved in the formation of gemcitabine-resistance. From the level of gene and protein expression, we further found that emodin decreased the expression of Bcl-2, increased the expression of Bax and cytochrome-C, this was most obvious in combination group suggesting that low concentration of emodin could reverse the increase of Bcl-2 and the decrease of Bax induced by resistance, but had no significant pro-apoptotic effect, and therefore enhanced the sensitivity of gemcitabine-resistant pancreatic cells to gemcitabine.

NF-κB is a family of ubiquitous transcription factors involving immunity, inflammation, regulation of cell growth, differentiation, apoptosis, and tumor metastasis. In recent reports, NF-κB is shown closely related to tumor resistance to chemotherapy. As previous studies show, down-regulation of anti-apoptotic protein is one of the mechanisms that NF-κB takes part in apoptosis and induces apoptosis (35). Banerjee *et al* have reported that NF-κB caused the resistance of pancreatic cancer through up-regulating the expression of anti-apoptotic proteins (XIAP, Bcl-xL, Survivin) (21). Another report shows that NF-κB induced the resistance of breast cancer by increasing the expression of anti-apoptotic protein Bcl-2 and decreasing the expression of pro-apoptotic protein Bax (36). Also, there are reports that NF-κB can overcome the chemotherapeutic resistance through down-regulating of expression of anti-apoptotic protein Bcl-2 family (37). Our study suggested that NF-κB participated in the formation of tumor resistance via multidrug resistance encoding protein P-gp and Bcl-2, with Bax that existed in mitochondrial apoptosis pathway. In this study we found that emodin reversed the gemcitabine resistance effect in pancreatic cancer, the action might be associating with down-regulation of NF-κB expression, and lowering the expression of P-gp and Bcl-2, increasing Bax expression.

In conclusion, emodin can effectively reverse the resistance effect of pancreatic cancer to gemcitabine. The potential mechanisms are 1) the decrease in the expression and function of P-gp, thus causing decrease of the efflux of drug and then increasing the intracellular drug concentration, thus the treatment effect was enhanced, 2) the down-regulation of Bcl-2 expression in mitochondrial apoptosis pathway and the up-regulation of Bax in mitochondrial pathway, followed by the occurrence of apoptosis.

## Figures and Tables

**Table I:** RT-PCR sequences of primers and the size of the sequences.

Gene	Sense primer	Antisense primer	PCR product (bp)
MDR-1	GAATCTGGAGGAAGACATGACC	TCCAATTTTGTCACCAATTCC	259
NF-κB	AGCACAGATACCACCAAGACCC	CCCACGCTGCTCTTCTATAGCAAC	300
Bcl-2	AGCCGGGAGAACAGGGTATG	ATCCAGGTGTGCATGCCG	549
Bax	ATGGCTGGGGAGACACCTGA	TGGGCGTCCCGAAGTAGGAA	394
Cyt-c	GCGTGTCCTTGGACTTAGAG	GGCGGCTGTGTAAGAGTATC	241
Caspase-9	GGTTCTGGAGGATTTGGTGA	GACAGCCGTGAGAGAGAATGA	325
Caspase-3	AGCAAACCTCAGGGAAACATT	GTCTCAATGCCACAGTCCAGT	309
GAPDH	AACGGATTTGGTCGTATTGGG	TCGCTCCTGGAAGATGGTGAT	216
